# Reducing gender disparities in post-total knee arthroplasty expectations through a decision aid

**DOI:** 10.1186/s12891-015-0473-x

**Published:** 2015-02-07

**Authors:** Elizabeth R Volkmann, John D FitzGerald

**Affiliations:** Division of Rheumatology, Department of Medicine, David Geffen School of Medicine, University of California, 1000 Veteran Avenue, Suite 32-59, Los Angeles, CA 90095 USA

**Keywords:** Osteoarthritis, Decision aid, Knee arthroplasty, Gender

## Abstract

**Background:**

Gender disparities in total knee arthroplasty utilization may be due to differences in perceptions and expectations about total knee arthroplasty outcomes. This study evaluates the impact of a decision aid on perceptions about total knee arthroplasty and decision-making parameters among patients with knee osteoarthritis.

**Methods:**

Patients with moderate to severe knee osteoarthritis viewed a video about knee osteoarthritis treatments options, including total knee arthroplasty, and received a personalized arthritis report. An adapted version of the Western Ontario and McMaster Universities Osteoarthritis Index was used to assess pain and physical function expectations following total knee arthroplasty before/after the intervention. These scores were compared to an age- and gender-adjusted means for a cohort of patients who had undergone total knee arthroplasty. Decision readiness and conflict were also measured.

**Results:**

At baseline, both men and women had poorer expectations about post-operative pain and physical outcomes compared with observed outcomes of the comparator group. Following the intervention, women’s mean age-adjusted expectations about post- total knee arthroplasty pain outcomes improved (Pre: 27.0; Post: 21.8 [p =0.08; 95% CI −0.7, 11.0]) and were closer to observed post-TKA outcomes; whereas men did not have a significant change in their pain expectations (Pre: 21.3; Post: 19.6 [p = 0.6; 95% CI −5.8, 9.4]). Women also demonstrated a significant improvement in decision readiness; whereas men did not. Both genders had less decision conflict after the intervention.

**Conclusions:**

Both women and men with osteoarthritis had poor estimates of total knee arthroplasty outcomes. Women responded to the intervention with more accurate total knee arthroplasty outcome expectations and greater decision readiness. Improving patient knowledge of total knee arthroplasty through a decision aid may improve medical decision-making and reduce gender disparities in total knee arthroplasty utilization.

**Electronic supplementary material:**

The online version of this article (doi:10.1186/s12891-015-0473-x) contains supplementary material, which is available to authorized users.

## Background

Gender disparities in total joint replacement utilization for osteoarthritis (OA) exist [1]. Numerous studies have demonstrated that the prevalence of osteoarthritis-related disability is higher among women than men [2,3]. Furthermore, women who undergo total knee arthroplasty (TKA) have more severe knee OA and greater functional disabilities than do men at the time of surgery [4]. Moreover, while TKA is reportedly underutilized by both genders [5], the degree of underutilization is three times greater in women than in men [1].

While several factors may explain these gender differences [6,7], some studies have suggested that women may not be as well informed about TKA as an option for knee OA compared with men [1,8]. In a population-based study of patients with moderate to severe knee osteoarthritis, only 33 percent of women versus 42 percent of men reported having ever discussed arthroplasty with a physician, and of these patients 19 percent of women and 26 percent of men had ever discussed arthroplasty with an orthopedic surgeon [1]. After adjustment for age and disease severity, women were still significantly less likely to have discussed arthroplasty with either a physician or with an orthopedic surgeon.

A more recent study found that physicians provide less medical information regarding TKA to woman compared with men, regardless of the physicians’ specific recommendation for TKA [8]. Less familiarity with TKA may lead to poorer understanding about potential outcomes and therefore greater reluctance to undergo surgery.

Patient decision aids may ameliorate short-comings in patient knowledge attributable to limitations in the current medical model. Decision aids provide patient-centered, evidenced-based information on treatment options [9]. Through presenting a balanced view of the treatment options and their associated risks and benefits, decision aids help to improve patient knowledge and may help reduce this gender disparity in TKA utilization. Indeed, our prior research in male veterans demonstrated that differences in expectations in TKA outcomes between African American and Caucasian men were significantly reduced after exposure to a decision aid designed to enhance informed decision-making [10].

To our knowledge, no studies have assessed the impact of a decision aid on perceptions of TKA in men and women with knee OA. The purpose of the present study was to examine the impact of exposure to a decision aid on changes in expectations of health outcomes following TKA and to evaluate decision-making parameters of the decision aid among men and women with knee OA.

## Methods

### Study population

Male and female patients were recruited prospectively using flyers posted in the lobby and clinical waiting areas at the Rheumatology and Internal Medicine clinics at the University of California, Los Angeles (UCLA). Patients contacted the research assistant and were screened using a computer-assisted survey described below.

Eligible participants were between 55–85 years of age, able to speak and read English, and had moderate to severe knee OA, defined as having a score of >39 on the Western Ontario and McMaster Universities Osteoarthritis Index (WOMAC) [1,11]. Exclusion criteria included equal to or more than 3 medical comorbidities as assessed by an adapted Charlson comorbidity index [12], or a single specific comorbidity, such as dementia, stroke with residual plegia or paresis, cancer (other than skin) and/or end-stage liver disease. To capture a more homogenous cohort, patients reporting a history of inflammatory arthritis, recent significant knee trauma, residence in a nursing home or prior hip or knee replacement surgery were also excluded.

The Institutional Review Board at UCLA approved this study (protocol # G05-07-012-01A). All participants provided written informed consent. Patients who participated were offered small monetary compensation.

### Study design

At the time of screening, each eligible patient completed baseline questionnaires and was invited to a group meeting to attend the study intervention. After the intervention, patients completed follow-up questionnaires.

### Intervention

The intervention included 2 parts. The first part was a 45-minute videotape created by the nonprofit Informed Medical Decision-Making (IMDM), which describes both medical and surgical (TKA) treatment options for knee OA (version 2) [13]. Along with explanations about knee OA pathogenesis and treatments, the video includes patient interviews discussing why they chose their particular treatment (either surgical or medical).

The IMDM video was developed by the foundation after a literature search was performed to provide the video with evidence-based information. Patient character and physician commentaries supplement graphic data presentations. Patient interviews were conducted to provide a descriptive experience of living with knee OA using patients who chose TKA and those who instead chose non-surgical management [13].

The second part of the intervention was a personalized arthritis report. Subjects were provided a graphic and narrative presentation of how their current symptoms compared to gender- and age-adjusted pre-operative mean pain and physical function scores for patients who had undergone TKA (See Additional files 1 and 2). Subjects were also shown the gender-, age- and baseline symptom- adjusted means (and confidence regions) for patients undergoing TKA at 1-, 6- and 12-months after their surgery. The principal investigator was available to assist with interpretation of the report as needed. For more detailed description of report, please refer to our prior publication [10].

### Primary outcomes

The primary outcome was change in post-TKA expected pain and physical function before and after the intervention in men and women. Expected post-TKA pain and functional outcomes were assessed by a previously validated, adapted version of the WOMAC [10] to capture patient expectations after TKA. Briefly, patients were asked to describe the outcomes that they expected for a typical TKA patient after *full recovery from surgery*. Three 5-point Likert response questions addressed pain. Eight 5-point Likert response questions assessed physical activities. All scores were rescaled (0–100), where a higher score reflected poorer function and more pain.

To compare patient pain and physical function post-operative expectations from this project to observed outcomes in reference dataset, our (age, baseline WOMAC score adjusted) mean expectations were compared with (age, baseline WOMAC score adjusted) mean observed outcomes from an external reference, stratified by gender. The Kinemax Study group [11] was used as the comparator cohort (United States patients only) because this study was large, prospective, observational studies of primary TKA outcomes whose investigators were gracious to provide data to be uses as an external reference.

### Secondary outcomes

To evaluate the impact of the intervention as a decision aid (on each gender group), decision-making measures were measured. Patient factual recall was also assessed to determine whether patients (male vs. female) attended to the video differently.

#### Decision-making measures

Decisional readiness and decision conflict are established measures of decision-making interventions. Both the baseline and post-intervention questionnaires assessed patient decision readiness and decision conflict about willingness to consider TKA [14]. Decision readiness was assessed using a validated single-item instrument [14]. Patients were asked, “If you were to make a decision about knee surgery TODAY, how ready would you be to make that decision?” Responses ranged from “not at all” to “very” on a 6-point Likert scale. Decision conflict was evaluated using a 16-question Decision Conflict Scale [15]. The instrument has been validated and can distinguish between patients ready to implement decisions and undecided patients.

#### Patient recall

To assess patient recall of the video, patients were given a questionnaire showing the patient-video characters' faces and asked whether they recalled the patient discussion, were influenced by the patient, and remembered whether the patient had had surgery or not.

### Statistical analysis

All statistical analyses were performed using the Statistical Package for Social Sciences (SPSS) 19.0 for Windows (SPSS, Inc., Chicago, Ill.). Repeated measures analysis of variance was used to assess the effect of gender on change in WOMAC-based expectation scores controlling for age. The study was powered at 80% (2-tailed alpha set = 0.05) to detect the minimal perceptible clinical difference of 10 units [16] for pre- vs. post-intervention WOMAC-based expectation estimates of pain and function scales assuming baseline standard deviation of 17 units for each scale. The target sample size was 122 participants. Gender stratified reported pain and physical function WOMAC post-operative adjusted means for the external dataset were standardized to reflect the age and (pre-operative) pain and physical function distribution from our study sample. Gender specific WOMAC means between our sample and the standardized external sample were subsequently compared using pooled standard errors and student’s t-tests.

## Results

### Baseline characteristics of patients

A total of 136 patients (58 men, 78 women) completed screening, and a total of 111 patients (41 men, 70 women) attended the intervention and completed the follow up questionnaire (See Table 1 for baseline characteristics of patients). While more women (90%) than men (71%) completed the intervention and post-group testing, there were no significant differences between patients who dropped out of the study and those who did not by age, WOMAC physical function or pain scores, or SF-36 physical function scores.

**Table 1:** Characteristics of patient participants* ^†^

	**Women (N = 70)**	**Men (N = 41)**
**Age (years)** *Mean (Standard deviation)*	72 (8.2)	70 (9.6)
**Marital status**		
Married	24 (34)	16 (39)
Divorced	18 (26)	11 (27)
Widowed	12 (17)	5 (12)
Other	16 (23)	9 (22)
**Education level**		
Completed college	25 (36)	14 (34)
Completed graduate school	21 (30)	13 (32)
**SF-36, Physical function** *Mean (Standard error)*	38 (25.1)	39 (17.9)
**WOMAC, Physical function** *Mean (Standard error)*	22 (15.9)	24 (17.3)
**WOMAC, Pain** *Mean (Standard error)*	25 (17.6)	24 (19.2)

*Values represent *N* (%), except where otherwise noted.

^†^There were no significant differences in characteristics by gender.

For the 111 patients at baseline (basis for all results reported below), women and men reported a similar OA severity as measured by WOMAC pain and physical functions scores and similar SF-36 physical function scores (Table 1).

### Patient knowledge of TKA

Prior to the intervention, 93% of patients (no gender difference) had heard of surgery as a treatment for knee OA. However, women in our sample were more likely to know someone who had TKA surgery than were men (78% versus 56%, respectively, p = 0.006). Among patients who knew someone who had surgery (n = 70), the respondents’ impressions were largely positive, with 68% rating surgery either quite helpful or very helpful (no gender differences).

### Baseline expected pain and physical function after TKA

**Figure 1:**
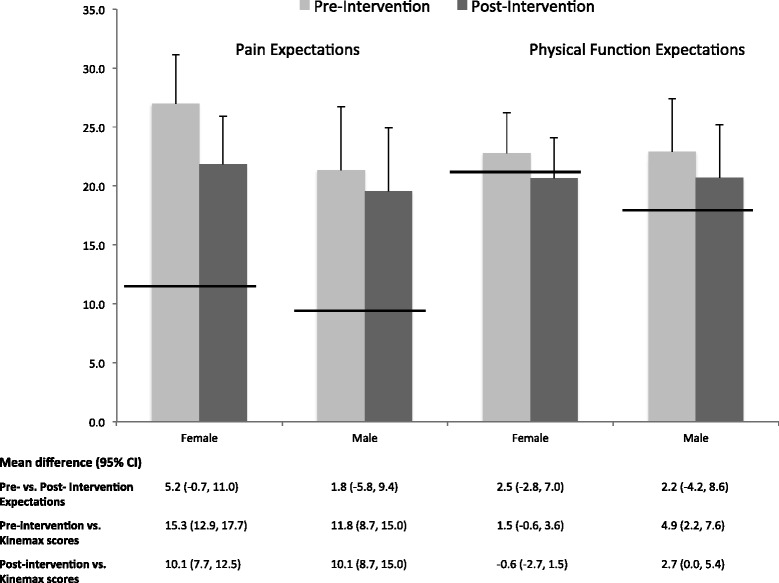
**Pre (light gray)- and post (dark gray)- intervention expectations on TKA outcomes versus reported 12-month post-TKA outcomes.** Vertical bars describe female (n = 71) and male (n = 40) scores (mean ± 95% confidence interval) on adapted WOMAC expectation instrument with higher scores representing greater pain and poorer physical function. Thick horizontal lines describe mean 12-month post-TKA pain and physical function scores by gender from the US cohort of the Kinemax study [11].

Patient expectations were assessed using the adapted WOMAC expectations instrument. Prior to the intervention, women had poorer age-adjusted mean post-TKA expectation pain scores at baseline than did men (27.0 versus 21.3, for women and men respectively; adjusted mean difference 5.3; p = 0.1; 95% CI −12.4, 1.2]), although this difference was not statistically significant. However, expectations about post-operative physical function were similar between the genders (22.8 versus 22.9, for women and men respectively; adjusted mean difference 0.1; p = 0.9; 95% CI −5.6, 5.8) (Figure 1).

Comparing our patients’ expectations about post-operative pain to the observed age-, gender- and pre-operative pain-adjusted outcomes from the Kinemax reference group, both women and men in our sample had significantly poorer pain expectations than the observed outcomes in the Kinemax. As noted above, for women in our group, expected WOMAC pain score was 27.0 versus 11.7 for the observed Kinemax outcomes (Adjusted mean difference 15.7; 95% CI 12.9, 17.7; p < 0.0001). Men in our group showed similarly poor expectations about expected post-operative pain versus observed Kinemax outcomes (21.3 vs. 9.5, respectively; Adjusted mean difference 11.8; p < 0.0001; 95% CI 8.9, 15.0) (Figure 1).

With respect to physical function, there were no significant differences in post-TKA estimates of physical function between the present cohort and the comparator cohort from the Kinemax study. At baseline, the post-TKA physical function score for women in our study was 22.8 compared with 21.3 for women from the Kinemax study (Adjusted mean difference 1.5; p = 0.4; 95% CI −0.6, 3.6). The post-TKA physical function score for men in our study was 22.9 compared with an 18.0 for men from the Kinemax study (Adjusted mean difference 4.9; p = 0.06; 95% CI 2.2, 7.6).

### Primary outcome: post-intervention expected pain and physical function after TKA

After the intervention, there was a trend for an improvement in women’s adjusted mean postoperative pain expectation score (Pre: 27.0 vs. Post: 21.8 [Adjusted mean difference 5.2; p =0.08; 95% CI −0.7, 11.0]). With respect to the Kinemax observed outcomes, after the educational intervention, women’s expectations were closer to reported post-TKA outcomes. However, men’s adjusted mean post-TKA pain score did not significantly change after the video (Pre: 21.3 vs. Post: 19.6, [Adjusted mean difference 1.8; p = 0.6; 95% CI −5.8, 9.4]), and the difference between the post-TKA pain scores for the study group men and Kinemax group men remained statistically significant (Adjusted mean difference 10.1; 95% CI 8.7, 15) (Figure 1).

Though not statistically significant, after the intervention, women and men’s mean physical function scores both improved (Figure 1). Women’s adjusted mean postoperative physical function scores improved from 22.8 to 20.7 (Adjusted mean difference 2.5; p = 0.4; 95% CI −2.8, 7.0). Men’s adjusted mean postoperative physical function scores improved from 22.9 to 20.7 (Adjusted mean difference 2.2; p = 0.5; 95% CI −4.2, 8.6). There were no significant differences in post-intervention adjusted mean physical function scores and Kinemax physical function scores for woman (Adjusted mean difference −0.6; 95% CI −2.7, 1.5) or for men (Adjusted mean difference 2.7; 95% CI 0, 5.4).

### Secondary outcomes: decision making measures and factual recall

#### Decision readiness

Prior to the intervention, 53.8% of women expressed that they were not at all ready to make a decision about whether to pursue TKA versus 42.1% on men (p = 0.7). After the intervention, women demonstrated greater decision readiness and (29.3% “Not at all ready…”; p = 0.01 compared with pre-intervention readiness). There was a trend for men to report greater decision readiness after the intervention (29.3% “Not at all ready…”; p = 0.9).

#### Decisional conflict

Decisional conflict represents the amount of uncertainty and discomfort with a decision. On a 0 to 100 scale, higher scores indicate greater conflict. Scores exceeding 37.5 are associated with indecision while scores lower than 25 are associated with implementing decisions. At baseline, there were no significant differences in decisional conflict (Mean ± SD) between men and women (39.8 ± 19.5 and 42.4 ± 18.2, for men and women, respectively) indicating high decisional conflict about the decision. After the intervention, women had significantly less decisional conflict; whereas for men, there was a trend for less decisional conflict. Women’s post-intervention score (Mean ± SD) was 31.6 ± 13.9, p <0.0001, and men’s post-intervention score was 32.3 ± 17.7, p = 0.06 (both p-values for comparison with respective pre-intervention scores).

#### Factual recall of the video

There was evidence that women attended to the video more closely. In the post-intervention survey, patients were quizzed about the surgical status (TKA versus non-surgical medical management) of the 6 patient characters in the video. During the video, the 6 characters had described their knee osteaoarthritis treatment in brief, approximately 5-minute vignettes. Three patient characters opted to have surgery, 3 had decided against surgery and chose non-surgical management. Women, more accurately recalled the video characters surgical status than did men. Women correctly identified the treatment status for the 6 characters 78% of the time compared with 62% for men (p = 0.03).

## Discussion

This is the first study, to our knowledge, to evaluate the impact of a patient decision aid on improving post-TKA expectations for pain and physical function between women and men. Based on prior literature about gender differences in TKA utilization [1] and reported delayed surgery for women [4], we had postulated that there would be baseline differences in post-operative expectations between the genders. We observed that women had poor expectations about pain outcomes (though inference testing only approached significance with a p-value of 0.1) than did men. However, we did not find any differences between the genders for expectations about post-operative physical function.

We further demonstrated that both male and female post-operative pain expectations were significantly poorer than outcomes among patients undergoing TKA and that females had differentially less accurate perceptions about TKA outcomes compared with men. The educational patient decision aid significantly improved post-TKA expectations regarding pain for women, with more accurate expectations about actual outcomes after the intervention. Men showed little change in pain expectations. Women also demonstrated improved decision readiness and less decisional conflict about TKA; whereas men only demonstrated a trend for improved decision readiness and less decision conflict about TKA.

One plausible explanation for these gender differences in response to the decision aid may be that women attended to the video better than men, as women demonstrated more accurate recall of facts from the video. Additional reasons may include greater baseline anxiety/fear about TKA for women compared with men. While the present study did not assess baseline fear about TKA, a prior study found that women had greater anxiety about undergoing surgery compared with men [17]. Another possible explanation is that women may indeed not be as well informed about TKA as an option for knee OA. By improving patient expectations (moving them closer post-intervention estimates), it is possible that the decision aid decreased women’s fear by presenting realistic information on risks associated with TKA resulting in improved decision readiness.

The present study has some important limitations. As described above, this was a cross-sectional convenience sample of patients with moderate to severe knee osteoarthritis. This sample may not be representative of the larger group of patients with moderate to severe knee osteoarthritis undecided about surgery. Selection for participation in this study may also not represent patients who have already decided to undergo TKA. Furthermore, the decision aid and personalized arthritis report are reliant on a certain degree of medical literacy. The patients in our sample were relatively well-educated, recruited from a tertiary care center, and therefore, the findings may not be reproducible in less educated populations where medical literacy may not be as proficient. However, we were able to demonstrate similar efficacy with the same intervention among a cohort of Veterans with knee osteoarthritis whose educational levels were significantly lower than our current gender study [10].

## Conclusion

These limitations notwithstanding, the present study demonstrated that a decision aid has the potential to improve post-TKA expectations, particularly for women’s expectations about post-operative pain outcomes. It is also reassuring that where physical function expectations were accurate; no change in expectations was noted for the intervention. Given that the majority of patients view pain relief as one of the most important reasons to have TKA surgery [18], clarifying expectations regarding post-TKA pain may lead to more informed decision making, which may help to reduce gender disparity in TKA utilization.

## Additional files

Additional file 1:
**Personalized arthritis report on pain.** Example of a personalized arthritis report describing how a male participant’s current pain symptoms compared to gender- and age-adjusted pre-operative mean pain score for patients who had undergone. TKA. Additional file 2:
**Personalized arthritis report on physical function.** Example of a personalized arthritis report describing how a male participant’s current physical function impairments compared to gender- and age-adjusted pre-operative mean physical function score for patients who had undergone TKA.

## Abbreviations

OA: Osteoarthritis
TKA: Total knee arthroplasty
UCLA: University of California, Los Angeles
WOMAC: Western Ontario and McMaster Universities Osteoarthritis Index
IMDM: Informed medical decision-making
SPSS: Statistical package for social sciences

## References

[1] Hawker GA, Wright JG, Coyte PC, Williams JI, Harvey B, Glazier R (2000). Differences between men and women in the rate of use of hip and knee arthroplasty. N Engl J Med.

[2] Blagojevic M, Jinks C, Jeffery A, Jordan KP (2010). Risk factors for onset of osteoarthritis of the knee in older adults: a systematic review and meta-analysis. Osteoarthritis Cartilage.

[3] O’Connor MI (2006). Osteoarthritis of the hip and knee: sex and gender differences. Orthop Clin North Am.

[4] Parsley BS, Bertolusso R, Harrington M, Brekke A, Noble PC (2010). Influence of gender on age of treatment with TKA and functional outcome. Clin Orthop Relat Res.

[5] Juni P, Low N, Reichenbach S, Villiger PM, Williams S, Dieppe PA (2010). Gender inequity in the provision of care for hip disease: population-based cross-sectional study. Osteoarthritis Cartilage.

[6] Borkhoff CM, Hawker GA, Wright JG (2011). Patient gender affects the referral and recommendation for total joint arthroplasty. Clin Orthop Relat Res.

[7] Change HJ, Mehta PS, Rosenberg A, Scrimshaw SC (2004). Concerns of patients actively contemplating total knee replacement: differences by race and gender. Arthritis Rheum.

[8] Borkhoff CM, Hawker GA, Kreder HJ, Glazier RH, Mahomed NN, Wright JG (2013). Influence of patients’ gender on informed decision making regarding total knee arthroplasty. Arthritis Care Res.

[9] Jayadev C, Khan T, Coulter A, Beard DJ, Price AJ (2012). Patient decision aids in knee replacement surgery. Knee.

[10] Weng HH, Kaplan RM, Boscardin WJ, Maclean CH, Lee IY, Chen W (2007). Development of a decision aid to address racial disparities in utilization of knee replacement surgery. Arthritis Rheum.

[11] Lingard EA, Katz JN, Wright EA, Sledge CB, Kinemax Outcomes Group (2004). Predicting the outcome of total knee arthroplasty. J Bone Joint Surg Am.

[12] Sangha O, Stucki G, Liang MH, Fossel AH, Katz JN (2003). The self-administered comorbidity questionnaire: a new method to assess comorbidity for clinical and health services research. Arthritis Rheum.

[13] Treatment choices for knee osteoarthritis. Foundation for Informed Medical Decision-Making; 2005. [http://www.informedmedicaldecisions.org/]

[14] O’Connor AM (1995). Validation of a decisional conflict scale. Med Decis Making.

[15] FitzGerald JD, Orav EJ, Lee TH, Marcantonio ER, Poss R, Goldman L (2004). Patient quality of life during the 12 months following joint replacement surgery. Arthritis Rheum.

[16] Ehrich EW, Davies GM, Watson DJ, Bolognese JA, Seidenberg BC, Bellamy N (2000). Minimal perceptible clinical improvement with the Western Ontario and McMaster Universities osteoarthritis index questionnaire and global assessments in patients with osteoarthritis. J Rheumatol.

[17] Karlson EW, Daltroy LH, Liang M, Eaton HE, Katz JN (1997). Gender differences in patient preferences may underlie differential utilization of elective surgery. Am J Med.

[18] Meneghini RM, Russo GS, Lieberman JR (2014). Modern perceptions and expectations regarding total knee arthroplasty. J Knee Surg.

