# Supplementary material for: Reducing gender disparities in post-total knee arthroplasty expectations through a decision aid
Source: BMC Musculoskelet Disord. 2015 Feb 7;16(1):16. doi: 10.1186/s12891-015-0473-x (PMC4328497; doi:10.1186/s12891-015-0473-x)

The following charts are meant to give you an understanding about:

- How your symptoms compare to patients with arthritis who decide to have knee replacement surgery and
- How you might do if you were to have knee replacement surgery.

Your PAIN SCORE is 27, which is the far left black bar on the graph below and the solid line that continues on to the graph on the opposite page.

The average U.S. male patient aged 65 and older scored **62**, which is the dark grey bar and the dashed black line that continues to the next page.

The average male patient your age undergoing knee surgery has a pain score of **32**, which is the light grey bar on the right.

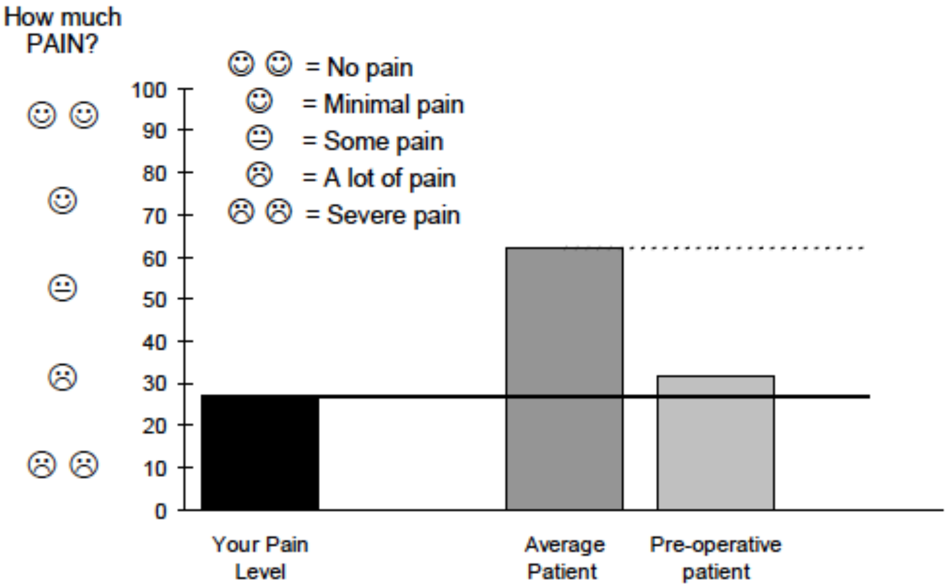


Now imagine you were to have surgery on January 1 of next year (marked on graph below by black vertical line).

At this point, the line showing YOUR PAIN SCORE splits into 3 lines and a shaded area to show how you might do during the year following surgery. If you had surgery, you would have a 50% chance of ending up anywhere within the shaded area. There is also a 50% chance you might end up either above or below the shaded area.

These predictions are based upon the actual results of patients who had knee surgery and were of similar age, same sex and same pre-operative PAIN SCORE that you currently have.

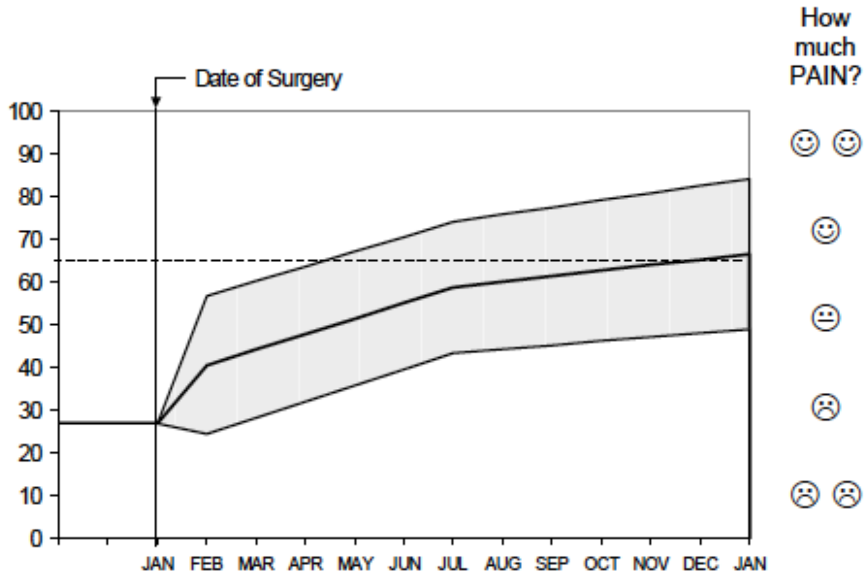

Supplement: Additional file 1: — Personalized arthritis report on pain. Example of a personalized arthritis report describing how a male participant’s current pain symptoms compared to gender- and age-adjusted pre-operative mean pain score for patients who had undergone. TKA. [file 12891_2015_473_MOESM1_ESM.pdf]
