# Supplementary material for: Reducing gender disparities in post-total knee arthroplasty expectations through a decision aid
Source: BMC Musculoskelet Disord. 2015 Feb 7;16(1):16. doi: 10.1186/s12891-015-0473-x (PMC4328497; doi:10.1186/s12891-015-0473-x)

Your PHYSICAL FUNCTION SCORE is 56, which is the left black bar on the graph below and the solid line that continues on to the graph on the opposite page.

The average U.S. male patient aged 65 and older scored **62**, which is the dark grey bar and dashed black line that continues on to the next page.

The average male patient your age undergoing knee surgery has a pain score of **34**, which is the light grey bar.

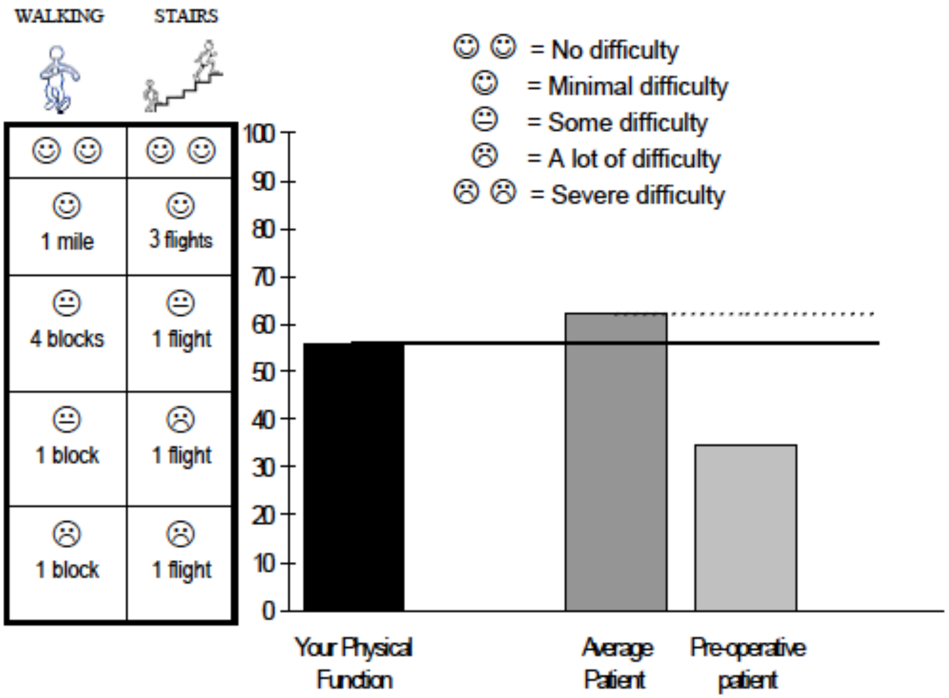

Again imagine you were to have surgery on January 1 of next year (marked on graph below by black vertical line).

At this point, the line showing YOUR PHYSICAL FUNCTION SCORE splits into 3 lines and a shaded area to show how you might do during the year following surgery. If you had surgery, you would have a 50% chance of ending up anywhere within the shaded area. There is also a 50% chance you might end up either above or below the shaded area.

These predictions are based upon the actual results of patients who had knee surgery and were of similar age, same sex and same pre-operative PHYSICAL FUNCTION SCORE that you currently have.

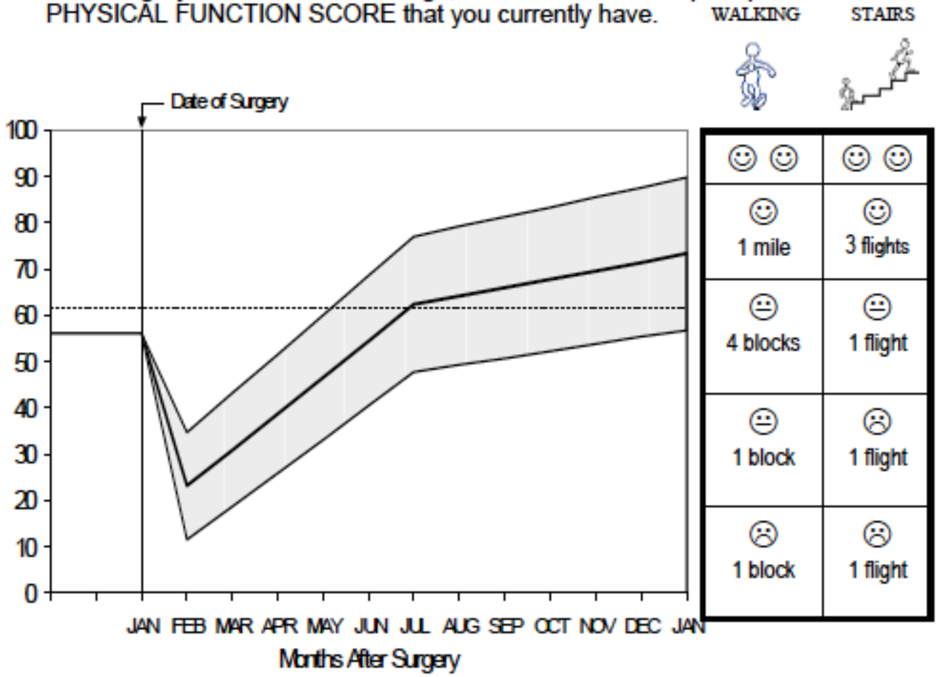

Supplement: Additional file 2: — Personalized arthritis report on physical function. Example of a personalized arthritis report describing how a male participant’s current physical function impairments compared to gender- and age-adjusted pre-operative mean physical function score for patients who had undergone TKA. [file 12891_2015_473_MOESM2_ESM.pdf]
